# Molecular pathology of intraductal papillary mucinous neoplasms of the pancreas: current understanding and perspectives on malignant progression

**DOI:** 10.1007/s00535-025-02328-7

**Published:** 2025-11-26

**Authors:** Yuki Makino, Kohki Oyama, Akiko Sagara, Fredrik Ivar Thege, Anirban Maitra

**Affiliations:** 1 Department of Gastroenterology and Hepatology, The University of Osaka Graduate School of Medicine, Suita, Osaka Japan; 2 Perlmutter Cancer Center, New York University Grossman School of Medicine, New York, NY USA; 3 Division of Surgical Oncology, The Ohio State University Wexner Medical Center, Columbus, OH USA

**Keywords:** IPMN, Pancreatic cancer, Molecular pathology, Multi-omics

## Abstract

Intraductal papillary mucinous neoplasms (IPMNs) of the pancreas are *bona fide* cystic precursor lesions to pancreatic ductal adenocarcinoma (PDAC), which is the cancer type with the most dismal prognosis. Since IPMNs are detectable by imaging, they offer a rare window of opportunity for early intervention for PDAC development. Despite their clinical visibility, the molecular pathogenesis of IPMNs remained incompletely understood, and no effective non-surgical therapeutic strategies have been established to date. In the past few decades, however, substantial progress has been made in elucidating their molecular pathology. Next-generation sequencing technologies demonstrated the comprehensive genetic mutation profile of IPMNs in the early 2010s. Elucidation of these mutation profiles enabled the establishment of genetically engineered mouse models, successfully recapitulating the natural development of human IPMNs and their progression to invasive cancer. Rapid evolution of “omics” technologies in recent years has facilitated the application of mass spectrometry, single-cell sequencing and spatial transcriptomics to IPMNs, significantly advancing our understanding of their pathophysiology. These techniques elucidated the changes in transcriptome, proteome, metabolome, microbiome, and tumor microenvironment associated with IPMN development and progression. This review summarizes current insights into the molecular and cellular landscapes of IPMN tumorigenesis, with particular emphasis on the mechanisms driving malignant progression.

## Introduction

Pancreatic ductal adenocarcinoma (PDAC) is one of the most lethal malignancies, with a steadily increasing incidence worldwide [1–3]. Despite advances in imaging and treatment modalities, its prognosis remains extremely poor, with a dismal 5-year survival rate of less than 10%. Globally, pancreatic cancer currently ranks among the highest causes of cancer-related mortality, and in the United States, it is projected to become the second leading cause of cancer death by 2030 [4]. This dire outcome is largely attributable to the fact that most patients are diagnosed at an advanced stage, when the disease has already progressed and surgical resection, the only potentially curative treatment, is no longer feasible. However, when detected and surgically resected at an early stage, this otherwise deadly disease can still be curable. Therefore, early detection and therapeutic intervention are essential for overcoming PDAC [5]. The key to this lies in the fact that pancreatic cancer arises from precursor lesions [1, 6, 7]. Two major precursors have been identified: pancreatic intraepithelial neoplasias (PanINs) and intraductal papillary mucinous neoplasms (IPMNs). PanINs are microscopic lesions that cannot be detected by conventional imaging techniques and are typically found only in histological specimens. In contrast, IPMNs are macroscopic cystic lesions which can be detected through radiologic imaging such as MRI or CT. This fundamental difference provides a crucial window of opportunity for clinical intervention, as IPMNs can be diagnosed and potentially treated before progressing to invasive carcinoma. Despite this potential, therapeutic options for IPMNs have remained largely limited to surgical resection [8–12]. There is currently no established non-surgical therapy for IPMN, underscoring the urgent need to better understand the molecular underpinnings of IPMN pathogenesis in order to develop molecular-targeted, non-surgical treatment strategies.

IPMNs were first described in Japan in the 1980s and have since been recognized as a distinct clinical and pathological entity [13]. However, for decades after its discovery, little progress had been made in elucidating its molecular characteristics. A major breakthrough occurred in the early 2010s with the advent of next-generation sequencing (NGS) technologies, which enabled comprehensive genomic analyses of IPMNs [14, 15]. Building on these findings, genetically engineered mouse models (GEMMs) were developed that recapitulate the genetic alterations and disease progression observed in human IPMNs. These autochthonous models are expected to serve as powerful tools for investigating the molecular mechanisms of IPMN development and progression, and evaluating novel therapeutic strategies in vivo. More recently, technological advances in multi-omics analyses have dissected the complex cellular and molecular landscape of IPMNs at unprecedented resolution, revealing key drivers of IPMN pathogenesis. These recent advances have transformed our understanding of IPMNs from a purely histopathological diagnosis to a molecularly defined precursor of pancreatic cancer with distinct biological subtypes and progression pathways. In this review, we provide a comprehensive overview of the recent progress in our understanding of the molecular pathology of IPMNs. We will provide an overview of the key genetic and epigenetic alterations, molecular signatures associated with disease progression, as well as the tumor microenvironment.

## Basics of IPMN pathology

IPMNs are grossly visible (> 1 cm) intraductal epithelial neoplasms, characterized by cystic dilatation of the pancreatic ducts and papillary growth of mucin-producing neoplastic epithelium [16–19]. IPMNs are classified based on their locations (main ducts (MD-IPMN), branch ducts (BD-IPMN), and mixed type), grade of dysplasia (Low-Grade (LG-IPMN), High-Grade (HG-IPMN)), or morphological subtypes (gastric, intestinal, or pancreatobiliary) [18]. A certain degree of concordance has been observed among these classification systems [9, 20, 21].

Gastric-type IPMNs are the most frequent subtype that resemble gastric foveolar or pyloric glands [9, 10, 12, 16, 18, 20, 21]. They consist of columnar cells with basally situated nuclei and prominent apical mucinous cytoplasm which express MUC5AC and MUC6 apomucins. They are typically low-grade, often arise in branch ducts, and are associated with favorable prognosis. Intestinal-type IPMNs resemble intestinal villous architecture composed of tall columnar epithelial cells which express MUC2, MUC5AC, and CDX2, a transcription factor that plays a crucial role in the development and maintenance of the intestinal epithelium [9, 10, 12, 16, 18, 20–23]. They usually develop from the main ducts and are commonly associated with a mucin-rich “colloid” carcinoma when they become invasive, and the latter tends to have a better prognosis than conventional “tubular” ductal adenocarcinoma. Pancreatobiliary-type IPMNs exhibit intricately branching, fern-like papillae composed of epithelial cells displaying nuclear enlargement, hyperchromasia, and moderately amphophilic cytoplasm, characterized by the expression of MUC1, MUC5AC, and MUC6 [9, 10, 12, 16, 18, 20, 21]. These are typically high-grade lesions, with a higher risk of progression to tubular adenocarcinoma associated with the poorest outcomes [24].

These subtypes are often observed to coexist within the same lesion, suggesting transitions between them. For example, gastric-type IPMNs can coexist with the pancreatobiliary type, which has led to the hypothesis that the latter is a high-grade variant of the gastric type [25]. Omori et al. reported morphological transition from gastric to intestinal type as well in 60% of intestinal-type IPMNs [22]. All histological subtypes express MUC5AC, a marker highly expressed in gastric mucosa. Gastric-type IPMNs harbor the fewest genetic alterations, and their genetic and epigenetic profiles resemble those of PanINs, consistent with low-grade features [26, 27]. Based on these findings, gastric-type lesions are currently suggested to be a common precursor for other non-gastric subtypes [28].

## Genetics of IPMNs

Introduction of NGS technology in the early 2010s identified comprehensive genetic alteration profiles in IPMNs, leading to the discovery of mutations in new genes including *GNAS* and *RNF43*, as well as previously known oncogenic *KRAS* mutations [14, 15, 29].

### *KRAS*

Similar to PanINs, *KRAS* mutations are the most common genetic alterations in IPMNs [14, 15, 29, 30]. Most studies have reported no significant difference in its frequency between LG- and HG-IPMNs [10]. The *KRAS* gene encodes a small GTPase involved in the RAS/MAPK signaling pathway. Activating mutations in codon 12 (such as G12D and G12V) are found in approximately 65–90% of IPMNs [14, 30]. These mutations lead to constitutive activation of the KRAS protein by impairing its GTPase activity, thereby driving persistent stimulation of cellular proliferation [31]. Pancreas-specific Kras^G12D^ expression is sufficient to develop PanIN-PDAC in mice [32]. Also, Kras^G12D^ expression is essential for PDAC maintenance [33]. Although the significance of targeting oncogenic KRAS has not yet been studied in IPMNs, the high frequency of alterations and presence in even low-grade IPMNs suggests that it is likely required for both tumor initiation and maintenance much like in non-cystic PDAC.

### *GNAS*

The second most frequently mutated gene is *GNAS*, which encodes the stimulatory alpha subunit of the G-protein (Gsα). Gsα mediates G protein-coupled receptor (GPCR) signaling by stimulating adenylate cyclase, increasing cyclic AMP (cAMP) production, and activating cAMP-dependent protein kinase A [34]. The most commonly identified *GNAS* mutations are located at codon 201 (R201C or R201H), observed in around two-thirds of IPMNs [14, 35]. These mutations inhibit GTPase activity which converts bound GTP to GDP and inactivate Gsα, leading to the constitutive activation of GPCR signaling [36]. *GNAS* mutations are typically absent in PanIN-PDAC pathway and specific to IPMNs [14, 35]. In the majority of LG-IPMNs, *GNAS* mutations are already present [14] and notably, can be detected in duodenal fluid samples even before IPMN lesions become visible on radiologic imaging [37]. Some studies reported better prognosis in IPMNs with *GNAS* mutations [38, 39]. Kawabata et al. functionally demonstrated that mutant *GNAS* limits tumor aggressiveness via attenuating NOTCH signaling [40]. These observations suggest that *GNAS* mutations, like *KRAS* mutations, are primarily involved in the early tumorigenesis of IPMNs rather than in their malignant progression [41].

### *RNF43*

*RNF43* mutations are the third most frequent observed in 10–30% of IPMNs [29, 30], and are mainly inactivating nonsense or frameshift mutations [42]. *RNF43* gene encodes a transmembrane RING-type E3 ubiquitin ligase. It promotes the ubiquitination and degradation of target proteins, particularly Frizzled receptors, thereby negatively regulating the Wnt signaling pathway [43, 44]. Because Wnt signaling pathway is essential for cell proliferation, differentiation, and tissue homeostasis, RNF43 protein is thought to play a tumor-suppressive role by suppressing Wnt activity [42]. In contrast to *KRAS* and *GNAS*, *RNF43* mutations are observed predominantly in HG-IPMNs (20–75% vs. 0–10% in LG- IPMNs) [15, 29]. Chang et al. reported that *RNF43* mutations were associated with a worse prognosis in patients with invasive IPMNs, suggesting that loss of *RNF43* has an independent tumor-promotive roles in IPMNs [38].

### *KLF4*

Krüppel-like factor 4 (KLF4) is a member of the zinc finger transcription factor family first discovered in 1996 [45]. KLF4 regulates not only key cellular processes such as proliferation, differentiation, and apoptosis, but also plays a role in the pathogenesis of inflammation and tumorigenesis [46]. However, the underlying mechanisms of its diverse physiological and pathological functions remain poorly understood, and its role in many tumor types remains controversial and ambiguous [46]. The *KLF4* gene has recently been proven to be frequently mutated in IPMNs by Fujikura et al. who identified *KLF4* mutations in the cyst fluid in more than half of IPMN cases tested [47]. The prevalence of *KLF4* mutations is significantly higher in LG-IPMNs compared with HG-IPMNs [47]. In PDAC mouse models, KLF4 knockdown suppresses the formation of acinar to ductal metaplasia (ADM) and PanIN formation, while KLF4 overexpression promoted them [48, 49]. Notably, KLF4 expression is significantly decreased in non-cystic PDAC tissues compared to normal pancreatic tissues [50]. KLF4 inhibits the proliferation, EMT, invasion and metastasis in established PDAC cell lines [46, 51–54]. Based on these findings, KLF4 might be involved in tumor initiation but may also have a suppressive role in IPMN progression [47].

Four major driver gene mutations (*KRAS, TP53, CDKN2A, SMAD4*), often referred to as “Big Four”, are often found in PDACs. In addition to *KRAS*, the remaining three have also been investigated in IPMNs. *TP53* is the canonical tumor suppressor critical for maintaining genomic stability through cell cycle arrest and apoptosis [55]. Although somatic mutations in *TP53*, particularly missense mutations within its DNA-binding domain, are rarely detected in LG-IPMNs, they are frequently observed in HG-IPMNs or IPMN-derived carcinomas (38–50%) [56]. The *CDKN2A* gene encodes p16INK4a and p14ARF that regulate cell cycle progression [57]. Tissue-based studies support a stepwise increase in *CDKN2A* inactivation through loss of heterozygosity (LOH) during IPMN progression (12.5% in LG-IPMNs, 33% in HG-IPMNs, and 100% in invasive carcinoma). Loss of p16 protein expression is significantly more frequent in HG-IPMNs (50–100%) than in LG-IPMNs (10–51%), reinforcing its role in malignant progression. The *SMAD4* gene encodes a nuclear transcription factor that mediates TGF-β signaling and regulates cell cycle inhibition. *SMAD4* gene mutations are more frequently observed in IPMN-related PDACs as compared to the co-existing IPMNs [29, 58]. Although *SMAD4* expression is typically preserved in non-invasive IPMNs regardless of dysplasia grade, its loss has been observed in 55–75% of invasive carcinomas derived from IPMNs [59]. Alterations in the “Big Four” genes are thus frequently observed during IPMN progression, suggesting their potential contribution to the malignant transformation.

Based on the genetic alterations, various strategies have been explored to guide clinical decision-making through NGS analysis of cyst fluid, in which DNA is released from neoplastic epithelial cells lining the pancreatic cysts. Pancreatic cyst fluid can be obtained through endoscopic ultrasound-guided fine-needle aspiration (EUS-FNA). The patterns of genetic alterations detected in cyst fluid, such as mutations in *KRAS*, *GNAS*, *RNF43*, and *KLF4*, are useful for differentiating IPMNs from other pancreatic cystic lesions, including mucinous cystic neoplasms (MCNs), serous cystic neoplasms (SCNs), and solid pseudopapillary neoplasms (SPNs) [60–63]. Cyst fluid genetic profiling has also been utilized for assessing malignant potential, with mutations in *TP53*, *SMAD4*, *CDKN2A*, *PTEN*, and *PIK3CA* serving as indicators of advanced neoplasia [60–63]. Molecular marker examination in cyst fluid can thus support the differential diagnosis or risk stratification of pancreatic cysts, although cyst fluid collection via EUS-FNA is currently indicated only when the results are likely to change clinical management [12].

## Epigenetic modifications in IPMNs

In addition to the genetic alterations discussed above, growing evidence has highlighted the critical role of epigenetic dysregulation in IPMNs. Epigenetic modifications include three main types: (1) DNA methylation dysregulation, (2) abnormal histone modifications and chromatin remodeling, (3) dysregulation of non-coding RNAs, including lncRNAs and miRNAs [64, 65]. Among them, the association between DNA methylation and IPMNs has been relatively well studied. DNA methylation is a key epigenetic mechanism that involves the addition of a methyl group to the fifth carbon of cytosine residues, primarily within CpG islands in gene promoter regions [66]. This modification can silence gene expression either by blocking transcription factor binding or by altering chromatin structure to a more condensed, repressive state. Several studies have demonstrated extensive hypermethylation in at least one CpG island in IPMNs [67, 68]. Cyst fluid, pancreatic juice, and tissue samples were used to investigate the contribution of DNA methylation to IPMN progression. Hata et al. found that high-grade dysplasia and invasive carcinoma could be distinguished from low-grade dysplasia by some selected markers, including methylated *SOX17* [69]. In pancreatic juice, aberrant methylation of *TFPI-2* was observed more frequently in PDACs or HG-IPMNs than in LG-IPMNs, indicating that its silencing is a late event associated with malignant transformation [70, 71]. Other than these genes, multiple genes have been reported to be implicated in methylation-driven progression of IPMNs including *CDKN2A*, *RASSF1A*, *EFEMP1*, *RPRM*, and *CDO1* [10, 72, 73]. Aberrant DNA methylation was thus suggested to represent a hallmark of IPMN progression.

The SWI/SNF complexes are ATP-dependent chromatin remodeling complexes that alter the structure and positioning of nucleosomes to regulate gene expression [74, 75]. SWI/SNF complexes are commonly recruited to regions enriched with histone H3 lysine 27 acetylation (H3K27ac), a marker of active transcription, where they collaborate with transcription factors to promote chromatin accessibility [75]. Mutations in genes encoding subunits of SWI/SNF chromatin-remodeling complexes are observed in nearly 25% of all cancer types [76, 77]. Based on the several studies demonstrating that the loss of SWI/SNF subunits such as *SMARCB1*, *ARID1A*, *SMARCA4*, or *PBRM1* contributes to the development of various tumor types, SWI/SNF complexes are considered to have tumor-suppressive functions [78–80]. SWI/SNF complexes suppress tumors mainly by aiding transcription factors and possibly by helping DNA repair [75]. Dal Molin et al. reported that the loss of expression of the SWI/SNF chromatin remodeling subunit BRG1, coded by SMARCA4 gene, is frequently observed in IPMNs [81]. Loss of BRG1 becomes more frequent along with the advancement of histological grade of dysplasia, suggesting that it is associated with the progression of IPMNs.

 Whereas several epigenetic modifications have been reported, only a few studies have analyzed chromatin structures themselves of IPMNs. Among those limited studies, Kato et al. performed ATAC-seq analysis in human organoids established from normal pancreatic ducts, IPMNs, IPMNs with an associated invasive carcinoma, and PDACs [82]. They identified the MNX1-HNF1B transcription factor axis that regulates these epigenetic changes and is essential for the growth of IPMN-derived organoids, suggesting that epigenomic alterations could serve as potential therapeutic targets in the treatment of IPMNs.

## Establishment of animal models of IPMNs based on genetic/epigenetic alterations in human IPMNs

Since the establishment of pancreas-specific Kras mutant mice (“KC” mice) or Kras/p53 mutant mice (“KPC” mice) in early 2000s, these models have been widely used to study the PanIN-PDAC pathogenesis [32, 83]. Meanwhile, there were no well-established IPMN mouse models that accurately reflect both the genetic landscape of human IPMNs and their progression to invasive PDAC. With the increasing understanding of the genetic and epigenetic alterations of IPMNs as discussed above, various GEMM models have been developed over the past decade [20]. These autochthonous models recapitulate key genetic events and spontaneous tumor development with an immunocompetent native microenvironment, enabling researchers to study the cellular and molecular pathogenesis of IPMNs.

In line with the high prevalence of *KRAS* mutations in human IPMNs, the majority of IPMN GEMMs employ the LSL-*Kras*^G12D^ allele in combination with pancreas-specific *Cre* recombinase expression [84]. In combination with pancreas-specific expression of mutant *Kras*, three groups have reported IPMN model mice by introducing mutant *Gnas*. Taki et al. were the first to report pancreas-specific *Kras*/*Gnas* double-mutant mice by combining CAG-LSL-*Gnas*^R201H^ and LSL-*Kras*^G12D^ alleles with Ptf1a-Cre recombinase [85]. Within five weeks, these mice developed cystic pancreatic tumors characterized by markedly dilated ducts lined with papillary dysplastic epithelium, closely resembling human IPMNs. These findings clearly demonstrate that mutant *Gnas,* in cooperation with mutant *Kras*, plays a crucial role in the development of IPMNs. Patra et al. generated mice with inducible expression of *Gnas*^R201C^ under the control of a doxycycline-responsive promoter (TetO-*Gnas*^R201C^), and crossed them with Ptf1a-Cre, Rosa26-LSL-rtTA, and LSL-*Kras*^G12D^ mice [36]. These mice were administered with doxycycline to induce *Gnas*^R201C^ expression at 4 weeks of age onwards and developed IPMN-like large cystic tumors that shortly require euthanasia. Ideno et al. also established mice harboring Ptf1a-Cre, Rosa26-LSL-rtTA, TetO-*Gnas*^R201C^, and LSL-*Kras*^G12D^ alleles, which have been referred to as “*Kras;Gnas* mice” [86]. Although the transgenes were identical to those used in the model developed by Patra et al., the timing of doxycycline administration was modified to start at eight weeks of age, and these mice developed IPMN-like lesions that eventually progressed to PDAC without any additional transgenes. Investigation of the PDAC lesions in the *Kras*;*Gnas* mice, however, revealed loss of nuclear expression of at least one of the three tumor suppressor proteins (p53, Smad4, p16) in the “Big Four”, suggesting that their inactivation is required for progression to invasive carcinoma. These GEMMs carrying *Kras* and *Gnas* mutations genetically recapitulate human IPMNs and phenotypically mimic the IPMN–PDAC progression pathway, making them valuable autochthonous models of IPMNs.

Apart from *Kras*/*Gnas* double-mutant models, loss of epithelial *RNF43* with mutant *Kras* also leads to the development of IPMNs that progress to PDAC [87, 88]. Zuo et al. demonstrated that overexpression of KLF4 caused the formation of IPMN-like cystic lesions in the presence of *Kras* mutation [89]. Bardeesy et al. focused on the dysregulation of the TGFβ–Smad pathway and demonstrated that the combination of *Kras*^G12D^ and *Smad4* deficiency resulted in the rapid development of pancreatic tumors resembling IPMNs [90]. von Figura et al. reported that loss of *Brg1* in combination with mutant *Kras* resulted in the development of IPMN-like cystic lesions that progress to PDACs [91]. Kimura et al. found that knockout of another SWI/SNF complex subunit *Arid1a* in *Kras*-mutant pancreatic ductal cells developed IPMNs and PDACs [92]. These findings suggest that SWI/SNF chromatin remodeling complex suppresses the formation of IPMNs in pancreatic ductal cells. Serine/Threonine Kinase 11 (STK11), also known as liver kinase B1 (LKB1), is a serine/threonine kinase that indirectly suppresses the activity of the mTOR complex 1 (mTORC1) and activates AMP-activated protein kinase (AMPK) [93]. *STK11* is a known tumor suppressor and mutated in 26.6% of IPMNs [94]. Collet L et al. reported that pancreatic duct cell-specific *Kras*^G12D^ mutation and inactivation of STK11 induced IPMN development in mice [95].

Based on the genetic/epigenomic alterations identified in human IPMNs, various GEMMs have thus been developed, demonstrating that these changes can directly induce IPMN formation. Whereas mutant *KRAS* alone can induce PanIN development as mentioned earlier, these additional gene mutations can drive the transition from PanINs to IPMNs by some mechanisms. Several studies reported that mutant *GNAS* extensively alters expression profiles of mucin genes in pancreatic ductal cells, which may determine the characteristic phenotype of IPMNs [40, 96, 97]. In doxycycline-inducible *Gnas* mutation models, upregulation of apomucin genes is observed, as well as the gastric pit and spasmolytic polypeptide-expressing metaplasia markers, which are the markers of gastric-type IPMNs, upon mutant *Gnas* induction [97]. *Gnas* mutation is thus functionally related to the early tumorigenesis of IPMNs. Other than *GNAS*, however, biological functions of the molecular alterations discussed above are poorly understood. It remains unknown how they drive IPMN development in *KRAS*-mutant pancreatic epithelial cells. Further functional studies are needed to clarify the pathophysiological mechanisms underlying the formation of the characteristic cystic neoplasms.

## Multi-omics approach to molecular footprints in epithelial cells during IPMN development and progression

Until recently, the studies on the molecular events underlying IPMN progression have largely been limited to genetic mutations and epigenomic alterations. In contrast, recent advances in single-cell and spatial technologies, as well as mass spectrometry (MS), have facilitated the application of multi-omics analyses to IPMNs. These techniques provide new insights into dynamic changes in gene and protein expression in tumor cells, as well as the tumor microenvironment, from the perspective of malignant progression. Although multi-omics studies in IPMNs are still quite limited, their recent advances are beginning to enable a more comprehensive understanding of IPMN molecular pathogenesis.

Li et al. performed scRNA-seq in surgically resected HG-IPMNs and IPMN-derived PDACs which demonstrated heterogeneous epithelial cell clusters as acinar-ductal cells, isthmus-pit cells, and PDAC-unique ductal cells [98]. Isthmus-pit cells highly expressed gastric isthmus-pit (GIP) signature (*Gkn1*, *Mucl3*, *Tff1*, and *Muc5ac*), which are characteristic of gastric-type IPMNs. These cells were enriched in IPMN samples, whereas PDAC samples had paucity of these cell types. In a subsequent scRNA-seq analysis as well, *Muc5ac* expression was found to decrease along with the progression from LG-IPMNs to HG-IPMNs and PDACs [99]. This finding is plausible, as gastric-type IPMNs are commonly associated with low-grade lesions and serve as precursor lesions of other histological subtypes and PDACs. Meanwhile, the molecular mechanisms that drive gastric-type IPMNs remained unclear. Using spatial transcriptomics, the transcription factor NKX6-2 was identified as a driver of gastric-differentiation [100]. *Nkx6-2* was one of the top differentially expressed transcripts in LG-IPMN compared to HG-IPMN/PDAC within tumor epithelial cell regions. *Nkx6-2*-positive spots were enriched for the gastric isthmus/pit gene signature compared with *Nkx6-2*-negative spots. Functionally, enforced expression of NKX6-2 upregulated gastric isthmus/pit gene signature and induced indolent characteristics in an IPMN-derived cell line, which resulted in the formation of IPMN-like glandular histology in the orthotopic injection model. Of note, NKX6-2 expression was found to be lost during progression to HG-IPMN/PDACs. These findings suggest that NKX6-2 promotes gastric differentiation and contributes to the early development of IPMNs, while its downregulation may be involved in their malignant progression. Another study of spatial transcriptomics in IPMNs by Agostini et al. also confirmed that *Nkx6-2* is a marker of gastric IPMNs [101].

Comprehensive gene expression analyses have revealed changes in gene expression signatures associated with the histological progression of IPMN. In three studies utilizing spatial transcriptomics, the HALLMARK gene sets were used to analyze signatures enriched in tumor epithelial cells during IPMN progression from low grade to high grade IPMNs and PDACs [101–103]. Notably, all three studies consistently demonstrated that the tumor necrosis factor (TNF)—NF-κB signature, which serves as a pivotal mediator of inflammatory responses [104], was enriched along with the advancement of the histological grade. This observation was also supported by the scRNA-seq analysis by Li et al. [98]. These findings implicate inflammation as a potential contributing factor in the progression of IPMNs.

Wang et al. examined proteins and glycoproteins from IPMN tissues, cyst fluid, and various types of normal samples using MS [105]. They demonstrated the alterations in glycosylation, which is a major protein modification that occurs several cancer types [106]. N-glycosylation attaches glycan, a chain of sugar molecules, to asparagine and is important for protein folding and stability. O-glycosylation links glycans to serine or threonine; this includes the O-GalNAc type, which is common in mucins and cancer, and O-GlcNAc type, which regulates intracellular signaling. A rarer form, C-glycosylation, connects glycans directly to tryptophan. In addition, some proteins are anchored to membranes through GPI-glycans. Wang et al. reported that glycan biosynthesis, particularly mucin-type O-glycans, was significantly enriched in IPMNs compared with the normal pancreas [105]. Moreover, several glycan biosynthesis–related proteins, including PLOD3, GXYLT1, UAP1, GNE, and B3GNT3, showed increased expression during IPMN progression. Nieminen et al. performed glycan profiling via matrix-assisted laser desorption-ionization time-of-flight mass spectrometry (MALDI-TOF–MS) in IPMN tissues and non-neoplastic pancreatic controls and demonstrated that N-glycan profiles were significantly different between invasive IPMNs and healthy pancreatic tissues [107]. The involvement of glycan alterations in the progression of IPMNs can be assumed from the fact that abnormal glycosylation leads to increased CA19-9, which is used as a biomarker for pancreatic cancer [108]. Alongside inflammation, metabolic alterations, particularly those involving glycan metabolism, may have critical implications for the mechanisms of IPMN progression. Therefore, the following sections will review the current evidence regarding the roles of metabolism and inflammation in IPMN pathogenesis.

## Metabolic alterations in IPMNs

Metabolic reprogramming is a hallmark of cancer, enabling tumor cells to meet increased bioenergetic and biosynthetic demands, resist oxidative stress, and adapt to nutrient-deprived tumor microenvironments [65]. Glycan metabolism, particularly the biosynthesis and remodeling of glycans, is tightly coupled to cellular energy metabolism. Among the metabolic pathways, glycolysis plays a central role, not only in ATP production but also in supplying precursors for glycosylation [109]. Specifically, the glycolytic intermediate fructose-6-phosphate serves as the entry point to the hexosamine biosynthetic pathway (HBP), which generates UDP-N-acetylglucosamine (UDP-GlcNAc), a critical donor substrate for both N-linked and O-linked glycosylation [110]. Through this link, fluctuations in glycolytic flux directly affect the supply of nucleotide sugars, thereby influencing glycan branching, extension, and sialylation. To date, several reports have highlighted the relationship between genetic alterations observed in IPMNs and glycolysis.

Alterations in glycolysis in IPMNs have been explored from the perspective of underlying genetic mutations. Although described in the setting of PDACs, mutant KRAS enhances glycolysis by upregulating glucose transporters and glycolytic enzymes [33]. Another study showed that mutant RAS protein induces the expression of type I cytokine receptor complexes in cancer cells that enable them to receive cytokine growth signals (IL-4 or IL-13) from tumor microenvironment [111]. These extrinsic signals activate JAK1-STAT6 signaling, leading to the activation of MYC signaling that drives glycolysis. *KRAS*^G12D^-mediated glycolysis enhancement is thus not only cell-autonomous but also dependent on the tumor microenvironment. In IPMNs, it has been reported that the induction of mutant *Gnas*, in addition to mutant *Kras*, further enhances glycolysis via cAMP/PKA-mediated activating phosphorylation of the key glycolysis-regulating enzyme PFKFB3 [97]. Consequently, enhanced glycolysis is a potential therapeutic vulnerability in pancreatic cancer cells with *Kras* and *Gnas* co-mutation. Glycan profiling performed by Trinh et al. identified an increase in LacdiNAc (GalNAcβ1-4GlcNAc), a unique disaccharide structure often seen in N-glycans in some cancer types, upon mutant *Gnas* induction on *Kras*-mutant pancreatic tumor cells [112]. Mutant *Gnas* induction also resulted in the loss of pro-tumorigenic Lewis antigens, which can contribute to the indolent tumor characteristics. Based on enhanced glycolysis in the presence of *Gnas* mutation-driven PKA signaling, reprogramming of glucose metabolism can be one way that mutant *Gnas* modulates the glycan profile in IPMNs [97, 112]. As mentioned above, *GNAS* mutation upregulates the expression of gastric pit markers as well as spasmolytic polypeptide-expressing metaplasia markers [97]. Intriguingly, it has been further observed that suppression of glycolysis led to a marked reduction in the expression of these markers [97]. These findings imply that glycolytic activity is necessary for the increased expression of gastric pit markers and spasmolytic polypeptide-expressing metaplasia markers. This phenomenon may result from both alterations in glycan structures and epigenetic mechanisms such as histone lactylation [113], but the underlying details remain poorly understood. However, enhancement of glycolysis may play a role in conferring IPMN-specific traits to neoplastic cells by mutant *GNAS*.

Aside from glycolysis, Patra et al. reported that mutant *GNAS* reprograms lipid metabolism in pancreatic tumor cells [36]. Notably, fatty acid oxidation was enhanced and lipid remodeling was induced (reduced mono‑ and di‑acyl glyceride levels). MALDI-MS imaging in IPMN/PDAC tissues demonstrated enhanced sulfatide metabolism as an early metabolic alteration in IPMNs that persists through invasive neoplasia [114]. It was functionally demonstrated to be a potential actionable vulnerability in IPMN-derived PDACs [114]. IPMNs are characterized by markedly increased mucin production, which imposes a high biosynthetic demand, particularly for nucleotide sugars and glycosylation processes. To accommodate this demand, it is plausible that metabolic reprogramming occurs as shown above, enabling tumor cells to upregulate these pathways. Abnormal glycosylation, coupled with altered lipid and sulfatide metabolism, likely promotes enhanced mucin synthesis and secretion, as glycan assembly relies on nucleotide sugar supply while lipid and sulfatide pathways support vesicle trafficking and membrane dynamics. Based on these insights into metabolic reprogramming, further studies are needed to develop novel strategies for the early detection, malignant potential assessment, and timely treatment of IPMNs by targeting their distinct metabolic alterations.

## Inflammation and IPMN progression

As discussed above, another gene expression signature suggested to be associated with IPMN progression was inflammation. Inflammation drives carcinogenesis and contributes to every stage of tumor formation [115]. The significance of inflammation in pancreatic carcinogenesis has long been recognized. Repeated cycles of inflammation, injury, and metaplasia/repair are thought to promote tumor formation. Indeed, long-standing chronic pancreatitis is a known risk factor for PDACs. In IPMNs as well, the association between inflammation and tumor progression has been suggested by several clinical or translational studies in addition to the aforementioned transcriptomic analyses. IPMN patients with a history of acute pancreatitis had a higher incidence of IPMN-derived carcinoma compared with those without acute pancreatitis [116]. IPMN patients with chronic pancreatitis-like findings in the background pancreatic parenchyma on endoscopic ultrasonography had a higher prevalence of invasive intraductal papillary mucinous carcinoma [117]. In analyses using cyst fluid, the concentration of prostaglandin E2 (PGE2) in pancreatic cyst fluid was shown to correlate with the grade of IPMNs [118]. PGE2, derived from arachidonic acid via cyclooxygenase enzymes, functions to enhance local inflammation. Furthermore, in patients with high-grade dysplasia or invasive carcinoma, the levels of an inflammatory cytokine IL-1β in cyst fluid were higher compared with those in patients with low- or intermediate-grade dysplasia [119]. Regarding the blood markers, several studies have reported that the neutrophil-to-lymphocyte ratio (NLR) is elevated in patients with high-grade dysplasia or invasive IPMNs [120–123]. In addition, the C-reactive protein to albumin ratio (CAR) and ferritin, a marker of both acute and chronic inflammation, have also been reported to be associated with increasing malignancy of IPMNs [124, 125].

Inflammation within tumors is profoundly influenced by the tumor microenvironment. Generally, tumor cells exist within a complex microenvironment composed of various non-malignant elements such as fibroblasts, endothelial cells, and infiltrating immune cells [126]. Recent studies also suggest that bacterial communities form an integral part of this tumor ecosystem. Together, these diverse cellular and microbial components interact to shape the tumor microenvironment, influencing cancer development and progression [127]. In the following sections, therefore, we provide an overview of the microbiome and the tumor microenvironment with a focus on their alterations during IPMN progression.

## Microbiome in IPMNs

The pancreas had been traditionally regarded as a sterile organ, based on the long-standing belief that the highly alkaline pancreatic juice, rich in proteolytic enzymes, creates an environment inhospitable to microbial survival [128]. However, the recent advances in 16S rRNA sequencing techniques demonstrated the presence of bacteria in the normal pancreas [129]. Since the association between intratumor bacteria and chemotherapy resistance was reported [130], clinical and pathological significance of intratumor microbiome has been investigated in PDACs [131]. In IPMNs, intratumor bacteria have been investigated in tumor tissues and cyst fluid. Gaiser et al. examined surgically collected cyst fluid and showed that bacterial 16S DNA copy number was increased in cyst fluid from cases with high-grade dysplasia or IPMN-derived cancer compared with non-IPMN pancreatic cystic neoplasms [132]. They also demonstrated the co-occurrence and enrichment of oral bacterial taxa including *Fusobacterium nucleatum* and *Granulicatella adiacens* in cyst fluid from high-grade dysplasia cases, suggesting that intracystic bacterial DNA testing might be useful for clinical management of IPMNs. Hosaka et al. examined surgically resected IPMN tissues and demonstrated increased proportions of *Proteobacteria* and *Fusobacteria* in invasive IPMNs than in noninvasive IPMNs [133]. These two studies are consistent in demonstrating that the abundance of *Fusobacterium* increases with progression in histological grade. *Fusobacterium* has been implicated in promoting intratumoral inflammation and tumor progression [134–136], and these findings suggest that its enrichment in IPMNs may also contribute to inflammation and disease progression.

Although some studies have demonstrated alterations in the microbiome, others have reported an absence of detectable microbiota or only minimal changes associated with IPMN progression [137, 138]. These discrepancies may stem from differences in the analyzed materials (cyst fluid versus tumor tissue), sample types (FFPE versus fresh-frozen), variations in sample collection and preservation methods, risks of contamination, particularly in the specimens obtained through endoscopy rather than sterile surgical procedures [137, 139]. In addition to the conventional 16S rRNA sequencing or shotgun metagenomics, novel techniques such as 2bRAD-M sequencing have recently been used [140], which enable analysis of low-biomass and host DNA–contaminated samples [141]. Given the substantial heterogeneity in sample types and analytical methodologies among studies, the implementation of rigorous negative controls and cautious data interpretation are considered indispensable to ensure the reliability and validity of microbiome analyses in IPMNs [137].

## Profiles of tumor microenvironment in IPMNs

Just as the alterations in the intrinsic features of cancer cells, changes in the surrounding microenvironment also play a crucial role in the progression of IPMNs. Pancreatic cancers are considered an immunologically “cold” tumor, characterized by low antigenicity, limited effector T-cell infiltration, and a profoundly immunosuppressive microenvironment with minimal responsiveness to immune checkpoint blockade and other immunotherapies. Similar to PDAC, the immune microenvironment of PanINs exhibits immunosuppressive features, with reduced CD8 + T cells and infiltration of immunosuppressive cells, such as tumor-associated macrophages, myeloid-derived suppressor cells (MDSCs), and regulatory T cells (Tregs). These features appear even in low-grade lesions and persist during progression to invasive cancer [142, 143].

Meanwhile, IPMNs show immune cell infiltration at an early stage [144]. During IPMN progression, CD4 T-cell levels remain relatively unchanged, whereas CD8 T cells decrease and macrophages and Tregs markedly increase [144–147]. Although these studies have primarily relied on immunohistochemical analyses, recent advances in transcriptomic technologies have enabled a more comprehensive characterization. In a series of six IPMN cases with different histological grades, scRNA seq was performed to investigate the alterations in the tumor microenvironment during disease progression [99]. This study revealed a reduction in cytotoxic T cells and B cells, accompanied by an increase in MDSCs, as IPMNs progressed from LG to HG lesions and ultimately to PDACs. Further, an expansion of the inflammatory cancer-associated fibroblast (iCAF) subpopulation in PDAC samples, a subset previously shown to promote immunosuppression, secrete growth factors or inflammatory cytokines, and facilitate pro-tumorigenic roles. In contrast, myofibroblastic CAFs (myCAFs), another CAF subpopulation characterized by higher α-SMA expression and lower levels of inflammatory cytokines and chemokines [148], were rarely detected in LG-IPMNs but became highly enriched in HG-IPMNs. These findings imply that the transition of fibroblasts toward a myCAF phenotype can arise as early as the noninvasive stage.

Furthermore, spatial transcriptomic analyses to investigate the molecular mechanisms of the IPMN microenvironment found that, compared with HG-IPMNs and PDACs, LG-IPMNs harbored a significantly higher proportion of plasma cells and mast cells in close proximity to the neoplastic epithelium [100]. Abundance of plasma cells suggests that humoral immunity might play an important role in immune surveillance during the early stages of IPMN progression, whereas this function diminishes along with the advancement to high-grade dysplasia and invasive cancer. Mast cells have been implicated in the development and progression of conventional PDAC, and their potential involvement in the early tumorigenesis has also been suggested [149]. Thus, comprehensive analyses using novel transcriptomic technologies have revealed the presence of previously overlooked immune cell populations, advancing our understanding of the tumor microenvironment. On the other hand, the decrease in T cells and increase in macrophages during IPMN progression, initially identified through traditional immunostaining [129–132], were also validated by spatial transcriptomics [150].

## Concomitant PDAC: an unresolved yet clinically significant challenge

One of the clinically important issues in IPMNs is their potential not only to progress to invasive carcinoma itself but also to give rise to a concomitant PDAC (cPDAC) at a separate site, a phenomenon known as “dual carcinogenesis” [151]. Since the first reports of cPDAC in 1997 [152, 153], most studies investigating cPDACs have been conducted in Japan [154]. However, in recent years, the risk of cPDAC has also been increasingly recognized in the United States as well [155–157]. A recent large prospective cohort study of Japanese patients with branch duct IPMNs revealed that the incidence of cPDAC was comparable to that of IPMN-derived carcinoma [158]. Notably, despite regular six-month follow-up, as many as 34% (13/38) of cPDACs developed in this cohort were unresectable, underscoring the clinical importance of managing not only IPMN-derived carcinoma but also cPDACs.

Oyama et al. reported that while tumor size and main pancreatic duct diameter were significant risk factors for IPMN-derived carcinoma, they were not associated with cPDACs, with advanced age being the only independent risk factor [159]. This finding suggests different mechanisms of carcinogenesis between IPMN-derived carcinoma and cPDACs. Ideno et al. demonstrated that GNAS–wild-type and gastric-type IPMNs were significantly associated with cPDAC [23, 160]. Oi et al. reported that IPMNs accompanying cPDAC tended to be non-intestinal, non-invasive, and branch-ducttype IPMNs, frequently harboring KLF4 mutations [161]. These findings collectively suggest the possibility that intrinsic characteristics of IPMN can contribute to the development of cPDACs, yet further studies are warranted to elucidate the underlying mechanisms. More recently, Oyama et al. identified intrapancreatic fat deposition as a novel risk factor for cPDACs [162]. Fatty infiltration of the pancreas can induce the secretion of adipokines such as adiponectin and leptin, proinflammatory cytokines, and chemokines, which promote carcinogenesis through inflammation, enhanced cell proliferation and migration, inhibition of apoptosis, creation of tumor-promoting microenvironment [163]. Notably, intrapancreatic fat deposition was not a risk factor for IPMN-derived carcinoma, further supporting the notion that distinct pathogenic mechanisms underlie these two cancer types associated with IPMNs. Future studies aimed at elucidating the molecular basis of these differences may lead to risk stratification and more tailored strategies for the prevention and surveillance of pancreatic carcinogenesis in patients with IPMNs.

Overall, the molecular and cellular pathogenesis of cPDAC remains largely unexplored. In the future, comprehensive multi-omics profiling of cPDACs is expected to elucidate the differences and relationships between cPDAC and coexisting IPMN, leading to a better understanding of their mechanisms of carcinogenesis and associated risk factors.

## Summary and future perspectives

**Fig. 1:**
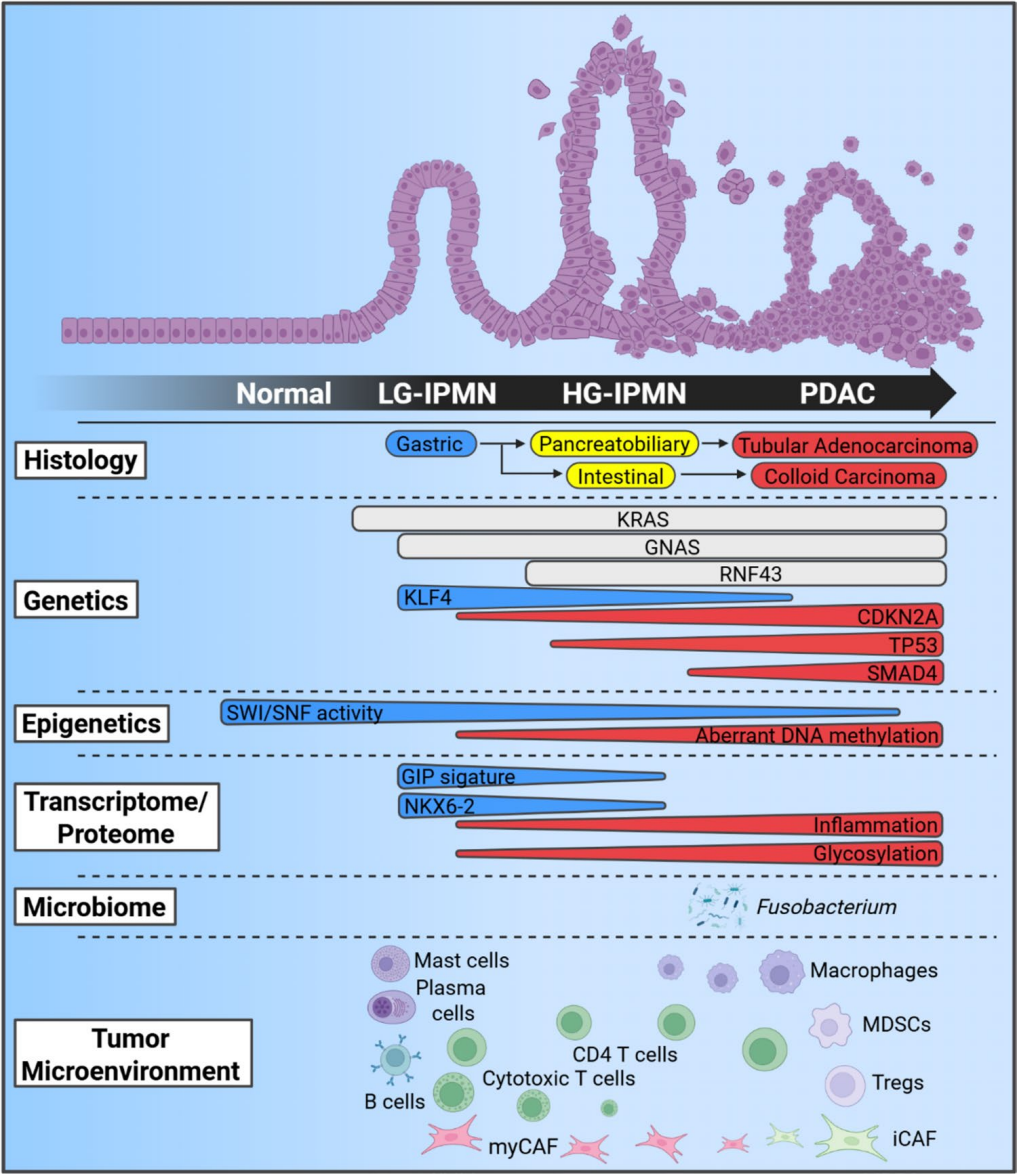
Molecular alterations in tumor cells and changes in the tumor microenvironment during IPMN progression. Created with BioRender.com

The investigation of IPMN pathogenesis was initiated by the identification of genetic alterations through NGS technologies. These technologies demonstrated that, like PDACs, the repertoire of driver mutations is relatively limited in IPMNs. However, behind this apparent simplicity lies a complex interplay of biological factors, including intratumoral heterogeneity, interactions with the microenvironment, and additional influences such as inflammation, metabolic alterations, and microbiome, all of which together shape a multifaceted disease state (Fig. 1). Recent advances in multi-omics technologies have provided valuable insights into these molecular and cellular dynamics.

There are two unmet clinical needs in IPMNs that should be addressed using these novel insights. The first is the optimization and personalization of patient management. Whereas current guidelines rely mainly on imaging findings and serum CA19-9 levels, integration with the multi-omics findings can enable more accurate risk stratification for malignancy to improve the decision-making regarding surveillance and surgical indications. For tumors like IPMNs where tissue acquisition through non-surgical approaches is difficult, liquid biopsy (cell-free DNA, extracellular vesicles) is considered a valuable tool for molecular analysis [164]. In particular, malignancy assessment using minimally invasive samples such as duodenal fluid or blood, in addition to cyst fluid, would be of great clinical value [164, 165]. The integration of imaging features and liquid biopsy markers may serve as a “radiogenomics signature” with potential utility for personalized medicine in IPMNs in the future.

The other challenge is the development of pharmacological therapies for IPMNs. Despite the identification of gene expression changes and evolving cellular populations during IPMN progression, studies addressing their functional significance remain quite limited. This seems to have been largely due to the lack of widely used animal models. With the development of GEMMs based on known driver mutations, functional analyses of individual molecules and cell types are now becoming feasible. These efforts are anticipated to uncover therapeutic targets capable of preventing their progression to pancreatic cancer, which will ultimately open the door to novel treatment strategies from the precancerous stage.

The recent advances in elucidating the molecular pathology of IPMNs, along with ongoing and future research, have the potential to profoundly transform the clinical management of this disease. The development of useful biomarkers could help avoid unnecessary imaging studies, potentially leading to a reduction in healthcare costs. By enabling therapeutic intervention and preventive strategies at the precancerous stage, it may become possible to halt or slow the progression to cancer. Such developments would represent a significant step forward in improving the prognosis of PDAC, which is one of the most aggressive and deadly cancer types.

## References

[1] Halbrook CJ, Lyssiotis CA, di Pasca Magliano M, Maitra A. Pancreatic cancer: advances and challenges. Cell. 2023;186(8):1729–54. 10.1016/j.cell.2023.02.014. 37059070 10.1016/j.cell.2023.02.014 PMC10182830

[2] Hosein AN, Dougan SK, Aguirre AJ, Maitra A. Translational advances in pancreatic ductal adenocarcinoma therapy. Nat Cancer. 2022;3(3):272–86. 10.1038/s43018-022-00349-2. 35352061 10.1038/s43018-022-00349-2

[3] Grossberg AJ, Chu LC, Deig CR, Fishman EK, Hwang WL, Maitra A, et al. Multidisciplinary standards of care and recent progress in pancreatic ductal adenocarcinoma. CA Cancer J Clin. 2020;70(5):375–403. 10.3322/caac.21626. 32683683 10.3322/caac.21626 PMC7722002

[4] Rahib L, Wehner MR, Matrisian LM, Nead KT. Estimated projection of US cancer incidence and death to 2040. JAMA Netw Open. 2021;4(4):e214708. 10.1001/jamanetworkopen.2021.4708. 33825840 10.1001/jamanetworkopen.2021.4708 PMC8027914

[5] Singhi AD, Koay EJ, Chari ST, Maitra A. Early Detection of Pancreatic Cancer: opportunities and challenges. Gastroenterology. 2019;156(7):2024–40. 10.1053/j.gastro.2019.01.259. 30721664 10.1053/j.gastro.2019.01.259 PMC6486851

[6] Faupel-Badger J, Kohaar I, Bahl M, et al. Defining precancer: a grand challenge for the cancer community. Nat Rev Cancer. 2024;24:792–809. 10.1038/s41568-024-00744-0. 39354069 10.1038/s41568-024-00744-0

[7] Stangis MM, Chen Z, Min J, Glass SE, Jackson JO, Radyk MD, et al. The hallmarks of precancer. Cancer Discov. 2024;14(4):683–9. 10.1158/2159-8290.CD-23-1550. 38571435 10.1158/2159-8290.CD-23-1550 PMC11170686

[8] Fonseca AL, Kirkwood K, Kim MP, et al. Intraductal papillary mucinous neoplasms of the pancreas: current understanding and future directions for stratification of malignancy risk. Pancreas. 2018;47:272–9. 10.1097/MPA.0000000000000999. 29424809 10.1097/MPA.0000000000000999 PMC5808987

[9] Moris D, Liapis I, Gupta P, et al. An overview for clinicians on intraductal papillary mucinous neoplasms (IPMNs) of the pancreas. Cancers (Basel). 2024. 10.3390/cancers16223825. 39594780 10.3390/cancers16223825 PMC11593033

[10] Corradi C, Gentiluomo M, Adsay V, Sainz J, Camisa PR, Wlodarczyk B, et al. Multi-omic markers of intraductal papillary mucinous neoplasms progression into pancreatic cancer. Semin Cancer Biol. 2025;109:25–43. 10.1016/j.semcancer.2024.12.005. 39733817 10.1016/j.semcancer.2024.12.005

[11] Gardner TB, Park WG, Allen PJ. Diagnosis and management of pancreatic cysts. Gastroenterology. 2024;167:454–68. 10.1053/j.gastro.2024.02.041. 38442782 10.1053/j.gastro.2024.02.041

[12] Ohtsuka T, Fernandez-Del Castillo C, Furukawa T, et al. International evidence-based Kyoto guidelines for the management of intraductal papillary mucinous neoplasm of the pancreas. Pancreatology. 2024;24:255–70. 10.1016/j.pan.2023.12.009. 38182527 10.1016/j.pan.2023.12.009

[13] Bishop MD, Wallace MB. Intraductal papillary mucinous neoplasms of the pancreas: should we side step the side branches? Clin Gastroenterol Hepatol. 2008;6(7):724–5. 10.1016/j.cgh.2008.04.022. 18602034 10.1016/j.cgh.2008.04.022

[14] Wu J, Matthaei H, Maitra A, et al. Recurrent GNAS mutations define an unexpected pathway for pancreatic cyst development. Sci Transl Med. 2011;3:92ra66. 10.1126/scitranslmed.3002543. 21775669 10.1126/scitranslmed.3002543 PMC3160649

[15] Wu J, Jiao Y, Dal Molin M, et al. Whole-exome sequencing of neoplastic cysts of the pancreas reveals recurrent mutations in components of ubiquitin-dependent pathways. Proc Natl Acad Sci U S A. 2011;108:21188–93. 10.1073/pnas.1118046108. 22158988 10.1073/pnas.1118046108 PMC3248495

[16] Wood LD, Adsay NV, Basturk O, et al. Systematic review of challenging issues in pathology of intraductal papillary mucinous neoplasms. Pancreatology. 2023;23:878–91. 10.1016/j.pan.2023.08.002. 37604731 10.1016/j.pan.2023.08.002

[17] Furukawa T. Mechanisms of development and progression of pancreatic neoplasms. Pathol Int. 2022;72(11):529–40. 10.1111/pin.13272. 36161420 10.1111/pin.13272 PMC9828726

[18] Pedro BA, Wood LD. Early neoplastic lesions of the pancreas: initiation, progression, and opportunities for precancer interception. J Clin Invest. 2025. 10.1172/JCI191937. 40662372 10.1172/JCI191937 PMC12259249

[19] Basturk O, Hong SM, Wood LD, Adsay NV, Albores-Saavedra J, Biankin AV, et al. A revised classification system and recommendations from the Baltimore Consensus Meeting for neoplastic precursor lesions in the pancreas. Am J Surg Pathol. 2015;39(12):1730–41. 10.1097/PAS.0000000000000533. 26559377 10.1097/PAS.0000000000000533 PMC4646710

[20] Li J, Wei T, Zhang J, et al. Intraductal papillary mucinous neoplasms of the pancreas: a review of their genetic characteristics and mouse models. Cancers (Basel). 2021. 10.3390/cancers13215296. 34771461 10.3390/cancers13215296 PMC8582516

[21] Hu Y, Jones D, Esnakula AK, et al. Molecular pathology of pancreatic cystic lesions with a focus on malignant progression. Cancers (Basel). 2024. 10.3390/cancers16061183. 38539517 10.3390/cancers16061183 PMC10969285

[22] Omori Y, Ono Y, Kobayashi T, et al. How does intestinal-type intraductal papillary mucinous neoplasm emerge? CDX2 plays a critical role in the process of intestinal differentiation and progression. Virchows Arch. 2020;477:21–31. 10.1007/s00428-020-02806-8. 32291497 10.1007/s00428-020-02806-8

[23] Ideno N, Ohtsuka T, Kono H, Fujiwara K, Oda Y, Aishima S, et al. Intraductal papillary mucinous neoplasms of the pancreas with distinct pancreatic ductal adenocarcinomas are frequently of gastric subtype. Ann Surg. 2013;258(1):141–51. 10.1097/SLA.0b013e31828cd008. 23532108 10.1097/SLA.0b013e31828cd008

[24] Distler M, Kersting S, Niedergethmann M, Aust DE, Franz M, Rückert F, et al. Pathohistological subtype predicts survival in patients with intraductal papillary mucinous neoplasm (IPMN) of the pancreas. Ann Surg. 2013;258(2):324–30. 10.1097/SLA.0b013e318287ab73. 23532107 10.1097/SLA.0b013e318287ab73

[25] Adsay NV, Conlon KC, Zee SY, et al. Intraductal papillary-mucinous neoplasms of the pancreas: an analysis of in situ and invasive carcinomas in 28 patients. Cancer. 2002;94:62–77. 10.1002/cncr.10203. 11815961 10.1002/cncr.10203

[26] Kobayashi T, Omori Y, Ono Y, et al. Pathways for the development of multiple epithelial types of intraductal papillary mucinous neoplasm of the pancreas. J Gastroenterol. 2021;56:581–92. 10.1007/s00535-021-01783-2. 33796937 10.1007/s00535-021-01783-2

[27] Liffers ST, Godfrey L, Frohn L, et al. Molecular heterogeneity and commonalities in pancreatic cancer precursors with gastric and intestinal phenotype. Gut. 2023;72:522–34. 10.1136/gutjnl-2021-326550. 35944927 10.1136/gutjnl-2021-326550 PMC9933174

[28] Noë M, Brosens LAA. Gastric- and intestinal-type IPMN: two of a kind? Virchows Arch. 2020;477:17–9. 10.1007/s00428-020-02827-3. 32399629 10.1007/s00428-020-02827-3

[29] Amato E, Molin MD, Mafficini A, Yu J, Malleo G, Rusev B, et al. Targeted next-generation sequencing of cancer genes dissects the molecular profiles of intraductal papillary neoplasms of the pancreas. J Pathol. 2014;233(3):217–27. 10.1002/path.4344. 24604757 10.1002/path.4344 PMC4057302

[30] Fischer CG, Beleva Guthrie V, Braxton AM, et al. Intraductal papillary mucinous neoplasms arise from multiple independent clones, each with distinct mutations. Gastroenterology. 2019;157:1123-37.e22. 10.1053/j.gastro.2019.06.001. 31175866 10.1053/j.gastro.2019.06.001 PMC6756950

[31] Singhal A, Li BT, O’Reilly EM. Targeting KRAS in cancer. Nat Med. 2024;30:969–83. 10.1038/s41591-024-02903-0. 38637634 10.1038/s41591-024-02903-0 PMC11845254

[32] Hingorani SR, Petricoin EF, Maitra A, et al. Preinvasive and invasive ductal pancreatic cancer and its early detection in the mouse. Cancer Cell. 2003;4:437–50. 10.1016/s1535-6108(03)00309-x. 14706336 10.1016/s1535-6108(03)00309-x

[33] Ying H, Kimmelman AC, Lyssiotis CA, et al. Oncogenic Kras maintains pancreatic tumors through regulation of anabolic glucose metabolism. Cell. 2012;149:656–70. 10.1016/j.cell.2012.01.058. 22541435 10.1016/j.cell.2012.01.058 PMC3472002

[34] Landis CA, Masters SB, Spada A, et al. GTPase inhibiting mutations activate the alpha chain of Gs and stimulate adenylyl cyclase in human pituitary tumours. Nature. 1989;340:692–6. 10.1038/340692a0. 2549426 10.1038/340692a0

[35] Furukawa T, Kuboki Y, Tanji E, et al. Whole-exome sequencing uncovers frequent GNAS mutations in intraductal papillary mucinous neoplasms of the pancreas. Sci Rep. 2011;1:161. 10.1038/srep00161. 22355676 10.1038/srep00161 PMC3240977

[36] Patra KC, Kato Y, Mizukami Y, et al. Mutant GNAS drives pancreatic tumourigenesis by inducing PKA-mediated SIK suppression and reprogramming lipid metabolism. Nat Cell Biol. 2018;20:811–22. 10.1038/s41556-018-0122-3. 29941929 10.1038/s41556-018-0122-3 PMC6044476

[37] Kanda M, Knight S, Topazian M, et al. Mutant GNAS detected in duodenal collections of secretin-stimulated pancreatic juice indicates the presence or emergence of pancreatic cysts. Gut. 2013;62:1024–33. 10.1136/gutjnl-2012-302823. 22859495 10.1136/gutjnl-2012-302823 PMC3893110

[38] Chang XY, Wu Y, Jiang Y, et al. Mutations in IPMN cases: a potential prognostic factor. Gastroenterol Res Pract. 2020;2020:1457452. 10.1155/2020/1457452. 32934653 10.1155/2020/1457452 PMC7479465

[39] Evans J, Shivok K, Chen HH, et al. Correlation of GNAS mutational status with oncologic outcomes in patients with resected intraductal papillary mucinous neoplasms. Cancers (Basel). 2025. 10.3390/cancers17040705. 40002298 10.3390/cancers17040705 PMC11852742

[40] Kawabata H, Ono Y, Tamamura N, et al. Mutant GNAS limits tumor aggressiveness in established pancreatic cancer via antagonizing the KRAS-pathway. J Gastroenterol. 2022;57:208–20. 10.1007/s00535-021-01846-4. 35018527 10.1007/s00535-021-01846-4

[41] Matthaei H, Wu J, Dal Molin M, Shi C, Perner S, Kristiansen G, et al. GNAS sequencing identifies IPMN-specific mutations in a subgroup of diminutive pancreatic cysts referred to as “incipient IPMNs.” Am J Surg Pathol. 2014;38(3):360–3. 10.1097/PAS.0000000000000117. 24525507 10.1097/PAS.0000000000000117 PMC3927228

[42] Huo X, Han W, Yang Z, Lu Y, Liu N, Hou H. RNF43 in cancer: molecular understanding and clinical significance in immunotherapy. J Gene Med. 2024;26(8):e3729. 10.1002/jgm.3729. 39146560 10.1002/jgm.3729

[43] Zebisch M, Xu Y, Krastev C, MacDonald BT, Chen M, Gilbert RJC, et al. Structural and molecular basis of ZNRF3/RNF43 transmembrane ubiquitin ligase inhibition by the Wnt agonist R-spondin. Nat Commun. 2013;4(1):2787. 10.1038/ncomms3787. 24225776 10.1038/ncomms3787 PMC3905715

[44] Koo BK, Spit M, Jordens I, Low TY, Stange DE, van de Wetering M, et al. Tumour suppressor RNF43 is a stem-cell E3 ligase that induces endocytosis of Wnt receptors. Nature. 2012;488(7413):665–9. 10.1038/nature11308. 22895187 10.1038/nature11308

[45] Garrett-Sinha LA, Eberspaecher H, Seldin MF, de Crombrugghe B. A gene for a novel zinc-finger protein expressed in differentiated epithelial cells and transiently in certain mesenchymal cells. J Biol Chem. 1996;271(49):31384–90. 10.1074/jbc.271.49.31384. 8940147 10.1074/jbc.271.49.31384

[46] He Z, He J, Xie K. KLF4 transcription factor in tumorigenesis. Cell Death Discov. 2023;9:118. 10.1038/s41420-023-01416-y. 37031197 10.1038/s41420-023-01416-y PMC10082813

[47] Fujikura K, Hosoda W, Felsenstein M, Song Q, Reiter JG, Zheng L, et al. <article-title update="added"> Multiregion whole-exome sequencing of intraductal papillary mucinous neoplasms reveals frequent somatic *KLF4* mutations predominantly in low-grade regions. Gut. 2021;70(5):928–39. 10.1136/gutjnl-2020-321217. 33028669 10.1136/gutjnl-2020-321217 PMC8262510

[48] Ganguly K, Krishn SR, Rachagani S, Jahan R, Shah A, Nallasamy P, et al. Secretory mucin 5AC promotes neoplastic progression by augmenting KLF4-mediated pancreatic cancer cell stemness. Cancer Res. 2021;81(1):91–102. 10.1158/0008-5472.CAN-20-1293. 33127746 10.1158/0008-5472.CAN-20-1293 PMC7990052

[49] Xie VK, Li Z, Yan Y, Jia Z, Zuo X, Ju Z, et al. DNA-methyltransferase 1 induces dedifferentiation of pancreatic cancer cells through silencing of Krüppel-like factor 4 expression. Clin Cancer Res. 2017;23(18):5585–97. 10.1158/1078-0432.CCR-17-0387. 28659310 10.1158/1078-0432.CCR-17-0387 PMC5600846

[50] Yang Z, Li D, Liu Z, et al. BIRC7 and KLF4 expression in benign and malignant lesions of pancreas and their clinicopathological significance. Cancer Biomark. 2016;17:437–44. 10.3233/CBM-160660. 27802195 10.3233/CBM-160660 PMC13020514

[51] Wei D, Kanai M, Jia Z, Le X, Xie K. <article-title update="added"> Krüppel-like factor 4 induces *p27Kip1* expression in and suppresses the growth and metastasis of human pancreatic cancer cells. Cancer Res. 2008;68(12):4631–9. 10.1158/0008-5472.CAN-07-5953. 18559508 10.1158/0008-5472.CAN-07-5953 PMC2481517

[52] Yan Y, Li Z, Kong X, Jia Z, Zuo X, Gagea M, et al. KLF4-mediated suppression of CD44 signaling negatively impacts pancreatic cancer stemness and metastasis. Cancer Res. 2016;76(8):2419–31. 10.1158/0008-5472.CAN-15-1691. 26880805 10.1158/0008-5472.CAN-15-1691 PMC4876033

[53] Guo K, Cui J, Quan M, Xie D, Jia Z, Wei D, et al. The novel KLF4/MSI2 signaling pathway regulates growth and metastasis of pancreatic cancer. Clin Cancer Res. 2017;23(3):687–96. 10.1158/1078-0432.CCR-16-1064. 27449499 10.1158/1078-0432.CCR-16-1064 PMC5253336

[54] Nagamine Y, Tomita M, Yamakuchi M, et al. Effects of Krüppel-like factor 4 mutation on the clinicopathological characteristics and its related protein expressions in intraductal papillary mucinous neoplasm of the pancreas. Pancreatology. 2025;25:1126–31. 10.1016/j.pan.2025.09.028. 41076409 10.1016/j.pan.2025.09.028

[55] Makino Y, Hikita H, Fukumoto K, Sung JH, Sakano Y, Murai K, et al. <article-title update="added">Constitutive activation of the tumor suppressor p53 in hepatocytes paradoxically promotes non–cell autonomous liver carcinogenesis. Cancer Res. 2022;82(16):2860–73. 10.1158/0008-5472.CAN-21-4390. 35696550 10.1158/0008-5472.CAN-21-4390 PMC9379366

[56] Kanda M, Sadakari Y, Borges M, et al. Mutant TP53 in duodenal samples of pancreatic juice from patients with pancreatic cancer or high-grade dysplasia. Clin Gastroenterol Hepatol. 2013;11:719-30.e5. 10.1016/j.cgh.2012.11.016. 23200980 10.1016/j.cgh.2012.11.016 PMC3600161

[57] Adam MP, Feldman J, Mirzaa GM, et al. GeneReviews. In: 1993.

[58] Omori Y, Ono Y, Tanino M, et al. Pathways of progression from intraductal papillary mucinous neoplasm to pancreatic ductal adenocarcinoma based on molecular features. Gastroenterology. 2019;156:647-61.e2. 10.1053/j.gastro.2018.10.029. 30342036 10.1053/j.gastro.2018.10.029

[59] Biankin AV, Biankin SA, Kench JG, et al. Aberrant p16(INK4A) and DPC4/Smad4 expression in intraductal papillary mucinous tumours of the pancreas is associated with invasive ductal adenocarcinoma. Gut. 2002;50:861–8. 10.1136/gut.50.6.861. 12010891 10.1136/gut.50.6.861 PMC1773240

[60] Singhi AD, McGrath K, Brand RE, et al. Preoperative next-generation sequencing of pancreatic cyst fluid is highly accurate in cyst classification and detection of advanced neoplasia. Gut. 2018;67:2131–41. 10.1136/gutjnl-2016-313586. 28970292 10.1136/gutjnl-2016-313586 PMC6241612

[61] Nikiforova MN, Wald AI, Spagnolo DM, Melan MA, Grupillo M, Lai Y-T, et al. A combined DNA/RNA-based next-generation sequencing platform to improve the classification of pancreatic cysts and early detection of pancreatic cancer arising from pancreatic cysts. Ann Surg. 2023;278(4):e789–97. 10.1097/sla.0000000000005904. 37212422 10.1097/SLA.0000000000005904 PMC10481930

[62] Paniccia A, Polanco PM, Boone BA, et al. Prospective, multi-institutional, real-time next-generation sequencing of pancreatic cyst fluid reveals diverse genomic alterations that improve the clinical management of pancreatic cysts. Gastroenterology. 2023;164:117-33.e7. 10.1053/j.gastro.2022.09.028. 36209796 10.1053/j.gastro.2022.09.028 PMC9844531

[63] Jones AR, Bardhi O, Polanco P, et al. Clinical utility of incorporating next-generation sequencing results in the management algorithm of pancreatic cysts. Gastrointest Endosc. 2025;102:223-32.e3. 10.1016/j.gie.2025.01.005. 39818341 10.1016/j.gie.2025.01.005

[64] Marei HE. Epigenetic regulators in cancer therapy and progression. NPJ Precis Oncol. 2025;9(1):206. 10.1038/s41698-025-01003-7. 40581650 10.1038/s41698-025-01003-7 PMC12206235

[65] Hanahan D. Hallmarks of cancer: new dimensions. Cancer Discov. 2022;12:31–46. 10.1158/2159-8290.CD-21-1059. 35022204 10.1158/2159-8290.CD-21-1059

[66] Fahrner JA, Eguchi S, Herman JG, et al. Dependence of histone modifications and gene expression on DNA hypermethylation in cancer. Cancer Res. 2002;62:7213–8. 12499261

[67] Sato N, Ueki T, Fukushima N, et al. Aberrant methylation of CpG islands in intraductal papillary mucinous neoplasms of the pancreas. Gastroenterology. 2002;123:365–72. 10.1053/gast.2002.34160. 12105864 10.1053/gast.2002.34160

[68] Hong SM, Omura N, Vincent A, Li A, Knight S, Yu J, et al. Genome-wide CpG island profiling of intraductal papillary mucinous neoplasms of the pancreas. Clin Cancer Res. 2012;18(3):700–12. 10.1158/1078-0432.CCR-11-1718. 22173550 10.1158/1078-0432.CCR-11-1718 PMC3271174

[69] Hata T, Dal Molin M, Hong SM, Tamura K, Suenaga M, Yu J, et al. Predicting the grade of dysplasia of pancreatic cystic neoplasms using cyst fluid DNA methylation markers. Clin Cancer Res. 2017;23(14):3935–44. 10.1158/1078-0432.CCR-16-2244. 28148542 10.1158/1078-0432.CCR-16-2244 PMC5511555

[70] Sato N, Parker AR, Fukushima N, et al. Epigenetic inactivation of TFPI-2 as a common mechanism associated with growth and invasion of pancreatic ductal adenocarcinoma. Oncogene. 2005;24:850–8. 10.1038/sj.onc.1208050. 15592528 10.1038/sj.onc.1208050

[71] Jiang P, Watanabe H, Okada G, Ohtsubo K, Mouri H, Tsuchiyama T, et al. Diagnostic utility of aberrant methylation of tissue factor pathway inhibitor 2 in pure pancreatic juice for pancreatic carcinoma. Cancer Sci. 2006;97(11):1267–73. 10.1111/j.1349-7006.2006.00308.x. 16965396 10.1111/j.1349-7006.2006.00308.x PMC11158502

[72] Fujiyama Y, Kumamoto Y, Nishizawa N, et al. Promoter DNA hypermethylation of the cysteine dioxygenase 1 (CDO1) gene in intraductal papillary mucinous neoplasm (IPMN). Ann Surg Oncol. 2020;27:4007–16. 10.1245/s10434-020-08291-2. 32144623 10.1245/s10434-020-08291-2

[73] Nakazato T, Suzuki Y, Tanaka R, et al. Effect of Reprimo Down-regulation on malignant transformation of intraductal papillary mucinous neoplasm. Pancreas. 2018;47:291–5. 10.1097/MPA.0000000000001002. 29401170 10.1097/MPA.0000000000001002

[74] Euskirchen G, Auerbach RK, Snyder M. SWI/SNF chromatin-remodeling factors: multiscale analyses and diverse functions. J Biol Chem. 2012;287(37):30897–905. 10.1074/jbc.R111.309302. 22952240 10.1074/jbc.R111.309302 PMC3438922

[75] Mittal P, Roberts CWM. The SWI/SNF complex in cancer - biology, biomarkers and therapy. Nat Rev Clin Oncol. 2020;17:435–48. 10.1038/s41571-020-0357-3. 32303701 10.1038/s41571-020-0357-3 PMC8723792

[76] Shain AH, Pollack JR. The spectrum of SWI/SNF mutations, ubiquitous in human cancers. PLoS ONE. 2013;8(1):e55119. 10.1371/journal.pone.0055119. 23355908 10.1371/journal.pone.0055119 PMC3552954

[77] Kadoch C, Hargreaves DC, Hodges C, Elias L, Ho L, Ranish J, et al. Proteomic and bioinformatic analysis of mammalian SWI/SNF complexes identifies extensive roles in human malignancy. Nat Genet. 2013;45(6):592–601. 10.1038/ng.2628. 23644491 10.1038/ng.2628 PMC3667980

[78] Mathur R, Alver BH, San Roman AK, Wilson BG, Wang X, Agoston AT, et al. ARID1A loss impairs enhancer-mediated gene regulation and drives colon cancer in mice. Nat Genet. 2017;49(2):296–302. 10.1038/ng.3744. 27941798 10.1038/ng.3744 PMC5285448

[79] Tomihara H, Carbone F, Perelli L, Huang JK, Soeung M, Rose JL, et al. Loss of ARID1A promotes epithelial-mesenchymal transition and sensitizes pancreatic tumors to proteotoxic stress. Cancer Res. 2021;81(2):332–43. 10.1158/0008-5472.CAN-19-3922. 33158812 10.1158/0008-5472.CAN-19-3922 PMC8728103

[80] Gu YF, Cohn S, Christie A, et al. Modeling renal cell carcinoma in mice: Cancer Discov. 2017;7:900–17. 10.1158/2159-8290.CD-17-0292. 28473526 10.1158/2159-8290.CD-17-0292 PMC5540776

[81] Dal Molin M, Hong SM, Hebbar S, Sharma R, Scrimieri F, de Wil RF, et al. Loss of expression of the SWI/SNF chromatin remodeling subunit BRG1/SMARCA4 is frequently observed in intraductal papillary mucinous neoplasms of the pancreas. Hum Pathol. 2012;43(4):585–91. 10.1016/j.humpath.2011.06.009. 21940037 10.1016/j.humpath.2011.06.009 PMC3246530

[82] Kato H, Tateishi K, Fujiwara H, et al. MNX1-HNF1B axis is indispensable for intraductal papillary mucinous neoplasm lineages. Gastroenterology. 2022;162:1272-87.e16. 10.1053/j.gastro.2021.12.254. 34953915 10.1053/j.gastro.2021.12.254

[83] Hingorani SR, Wang L, Multani AS, Combs C, Deramaudt TB, Hruban RH, et al. Trp53R172H and KrasG12D cooperate to promote chromosomal instability and widely metastatic pancreatic ductal adenocarcinoma in mice. Cancer Cell. 2005;7(5):469–83. 10.1016/j.ccr.2005.04.023. 15894267 10.1016/j.ccr.2005.04.023

[84] Jackson EL, Willis N, Mercer K, Bronson RT, Crowley D, Montoya R, et al. Analysis of lung tumor initiation and progression using conditional expression of oncogenic K-ras. Genes Dev. 2001;15(24):3243–8. 10.1101/gad.943001. 11751630 10.1101/gad.943001 PMC312845

[85] Taki K, Ohmuraya M, Tanji E, Komatsu H, Hashimoto D, Semba K, et al. GNAS(R201H) and Kras(G12D) cooperate to promote murine pancreatic tumorigenesis recapitulating human intraductal papillary mucinous neoplasm. Oncogene. 2016;35(18):2407–12. 10.1038/onc.2015.294. 26257060 10.1038/onc.2015.294

[86] Ideno N, Yamaguchi H, Ghosh B, et al. GNAS. Gastroenterology. 2018;155:1593-607.e12. 10.1053/j.gastro.2018.08.006. 30142336 10.1053/j.gastro.2018.08.006 PMC6219919

[87] Hosein AN, Dangol G, Okumura T, et al. Loss of Rnf43 accelerates Kras-mediated neoplasia and remodels the tumor immune microenvironment in pancreatic adenocarcinoma. Gastroenterology. 2022;162:1303–18. 10.1053/j.gastro.2021.12.273. 34973294 10.1053/j.gastro.2021.12.273 PMC8934289

[88] Zhou X, Sun Z, Zhang M, Qu X, Yang S, Wang L, et al. Deficient Rnf43 potentiates hyperactive Kras-mediated pancreatic preneoplasia initiation and malignant transformation. Anim Models Exp Med. 2022;5(1):61–71. 10.1002/ame2.12203. 10.1002/ame2.12203 PMC8879633 35229994

[89] Zuo X, Wang L, Liu Y, et al. Dysregulated KLF4 expression plays a pivotal role in the pathogenesis of pancreatic intraductal papillary mucinous neoplasms. Gut. 2025;74:327–9. 10.1136/gutjnl-2024-332255. 38969489 10.1136/gutjnl-2024-332255 PMC11874310

[90] Bardeesy N, Cheng KH, Berger JH, et al. Smad4 is dispensable for normal pancreas development yet critical in progression and tumor biology of pancreas cancer. Genes Dev. 2006;20:3130–46. 10.1101/gad.1478706. 17114584 10.1101/gad.1478706 PMC1635148

[91] von Figura G, Fukuda A, Roy N, et al. The chromatin regulator Brg1 suppresses formation of intraductal papillary mucinous neoplasm and pancreatic ductal adenocarcinoma. Nat Cell Biol. 2014;16:255–67. 10.1038/ncb2916. 24561622 10.1038/ncb2916 PMC4684081

[92] Kimura Y, Fukuda A, Ogawa S, et al. ARID1A maintains differentiation of pancreatic ductal cells and inhibits development of pancreatic ductal adenocarcinoma in mice. Gastroenterology. 2018;155:194-209.e2. 10.1053/j.gastro.2018.03.039. 29604291 10.1053/j.gastro.2018.03.039

[93] Shackelford DB, Shaw RJ. The LKB1-AMPK pathway: metabolism and growth control in tumour suppression. Nat Rev Cancer. 2009;9:563–75. 10.1038/nrc2676. 19629071 10.1038/nrc2676 PMC2756045

[94] Omori Y, Ono Y, Morikawa T, Motoi F, Higuchi R, Yamamoto M, et al. Serine/Threonine kinase 11 plays a canonical role in malignant progression of *KRAS*-mutant and *GNAS*-wild-type intraductal papillary mucinous neoplasms of the pancreas. Ann Surg. 2023;277(2):e384–95. 10.1097/SLA.0000000000004842. 33914475 10.1097/SLA.0000000000004842

[95] Collet L, Ghurburrun E, Meyers N, et al. <article-title update="added">*Kras* and *Lkb1* mutations synergistically induce intraductal papillary mucinous neoplasm derived from pancreatic duct cells. Gut. 2020;69:704–14. 10.1136/gutjnl-2018-318059. 31154393 10.1136/gutjnl-2018-318059

[96] Komatsu H, Tanji E, Sakata N, Aoki T, Motoi F, Naitoh T, et al. A *GNAS* mutation found in pancreatic intraductal papillary mucinous neoplasms induces drastic alterations of gene expression profiles with upregulation of mucin genes. PLoS ONE. 2014;9(2):e87875. 10.1371/journal.pone.0087875. 24498386 10.1371/journal.pone.0087875 PMC3912139

[97] Makino Y, Rajapakshe KI, Chellakkan Selvanesan B, Okumura T, Date K, Dutta P, et al. Metabolic reprogramming by mutant *GNAS* creates an actionable dependency in intraductal papillary mucinous neoplasms of the pancreas. Gut. 2024;74(1):75–88. 10.1136/gutjnl-2024-332412. 39277181 10.1136/gutjnl-2024-332412 PMC12014225

[98] Li J, Wei T, Ma K, Zhang J, Lu J, Zhao J, et al. Single-cell RNA sequencing highlights epithelial and microenvironmental heterogeneity in malignant progression of pancreatic ductal adenocarcinoma. Cancer Lett. 2024;584:216607. 10.1016/j.canlet.2024.216607. 38246225 10.1016/j.canlet.2024.216607

[99] Bernard V, Semaan A, Huang J, San Lucas FA, Mulu FC, Stephens BM, et al. Single-cell transcriptomics of pancreatic cancer precursors demonstrates epithelial and microenvironmental heterogeneity as an early event in neoplastic progression. Clin Cancer Res. 2019;25(7):2194–205. 10.1158/1078-0432.CCR-18-1955. 30385653 10.1158/1078-0432.CCR-18-1955 PMC6445737

[100] Sans M, Makino Y, Min J, et al. Spatial transcriptomics of intraductal papillary mucinous neoplasms of the pancreas identifies NKX6-2 as a driver of gastric differentiation and indolent biological potential. Cancer Discov. 2023;13:1844–61. 10.1158/2159-8290.CD-22-1200. 37285225 10.1158/2159-8290.CD-22-1200 PMC10880589

[101] Agostini A, Piro G, Inzani F, et al. Identification of spatially-resolved markers of malignant transformation in intraductal papillary mucinous neoplasms. Nat Commun. 2024;15:2764. 10.1038/s41467-024-46994-2. 38553466 10.1038/s41467-024-46994-2 PMC10980816

[102] Iyer MK, Shi C, Eckhoff AM, et al. Digital spatial profiling of intraductal papillary mucinous neoplasms: toward a molecular framework for risk stratification. Sci Adv. 2023;9:eade4582. 10.1126/sciadv.ade4582. 36930707 10.1126/sciadv.ade4582 PMC10022906

[103] Iyer MK, Fletcher AA, Okoye JO, Shi C, Chen F, Kanu EN, et al. Spatial transcriptomics of intraductal papillary mucinous neoplasms reveals divergent indolent and malignant states. Clin Cancer Res. 2025;31(9):1796–808. 10.1158/1078-0432.CCR-24-1529. 39969959 10.1158/1078-0432.CCR-24-1529 PMC12045729

[104] Liu T, Zhang L, Joo D, et al. NF-κB signaling in inflammation. Signal Transduct Target Ther. 2017;2:17023. 10.1038/sigtrans.2017.23. 29158945 10.1038/sigtrans.2017.23 PMC5661633

[105] Wang Y, Lih TM, Lee JW, et al. Multi-omic profiling of intraductal papillary neoplasms of the pancreas reveals distinct patterns and potential markers of progression. Cancer Cell. 2025. 10.1016/j.ccell.2025.08.001. 40882635 10.1016/j.ccell.2025.08.001 PMC12482137

[106] He M, Zhou X, Wang X. Glycosylation: mechanisms, biological functions and clinical implications. Signal Transduct Target Ther. 2024;9:194. 10.1038/s41392-024-01886-1. 39098853 10.1038/s41392-024-01886-1 PMC11298558

[107] Nieminen H, Nummela P, Satomaa T, et al. N-glycosylation in non-invasive and invasive intraductal papillary mucinous neoplasm. Sci Rep. 2023;13:13191. 10.1038/s41598-023-39220-4. 37580349 10.1038/s41598-023-39220-4 PMC10425445

[108] Engle DD, Tiriac H, Rivera KD, et al. The glycan CA19-9 promotes pancreatitis and pancreatic cancer in mice. Science. 2019;364:1156–62. 10.1126/science.aaw3145. 31221853 10.1126/science.aaw3145 PMC6705393

[109] Skiba NP, Bae H, Hamm HE. Mapping of effector binding sites of transducin alpha-subunit using G alpha t/G alpha i1 chimeras. J Biol Chem. 1996;271:413–24. 10.1074/jbc.271.1.413. 8550597 10.1074/jbc.271.1.413

[110] Hart GW. Nutrient regulation of signaling and transcription. J Biol Chem. 2019;294(7):2211–31. 10.1074/jbc.AW119.003226. 30626734 10.1074/jbc.AW119.003226 PMC6378989

[111] Dey P, Li J, Zhang J, et al. Oncogenic KRAS-driven metabolic reprogramming in pancreatic cancer cells utilizes cytokines from the tumor microenvironment. Cancer Discov. 2020;10:608–25. 10.1158/2159-8290.CD-19-0297. 32046984 10.1158/2159-8290.CD-19-0297 PMC7125035

[112] Trinh VQ, Ankenbauer KE, Torbit SM, et al. Mutant GNAS drives a pyloric metaplasia with tumor suppressive glycans in intraductal papillary mucinous neoplasia. bioRxiv. 2025. 10.1101/2024.02.25.581948 10.1016/j.celrep.2025.116684 PMC12860476 41370125

[113] Ren H, Tang Y, Zhang D. The emerging role of protein L-lactylation in metabolic regulation and cell signalling. Nat Metab. 2025;7:647–64. 10.1038/s42255-025-01259-0. 40175761 10.1038/s42255-025-01259-0

[114] Chen Y, Ballarò R, Sans M, Thege FI, Zuo M, Dou R, et al. Long-chain sulfatide enrichment is an actionable metabolic vulnerability in intraductal papillary mucinous neoplasm (IPMN)-associated pancreatic cancers. Gut. 2025;74(10):1638–52. 10.1136/gutjnl-2025-335220. 40268349 10.1136/gutjnl-2025-335220 PMC13352527

[115] Greten FR, Grivennikov SI. Inflammation and cancer: triggers, mechanisms, and consequences. Immunity. 2019;51:27–41. 10.1016/j.immuni.2019.06.025. 31315034 10.1016/j.immuni.2019.06.025 PMC6831096

[116] Tsutsumi K, Ohtsuka T, Oda Y, et al. A history of acute pancreatitis in intraductal papillary mucinous neoplasms of the pancreas is a potential predictive factor for malignant papillary subtype. Pancreatology. 2010;10:707–12. 10.1159/000320696. 21242711 10.1159/000320696

[117] Takenaka M, Masuda A, Shiomi H, Yagi Y, Zen Y, Sakai A, et al. Chronic pancreatitis finding by endoscopic ultrasonography in the pancreatic parenchyma of intraductal papillary mucinous neoplasms is associated with invasive intraductal papillary mucinous carcinoma. Oncology. 2017;93(1):61–8. 10.1159/000481232. 29258092 10.1159/000481232

[118] Yip-Schneider MT, Carr RA, Wu H, et al. Prostaglandin E. J Am Coll Surg. 2017;225:481–7. 10.1016/j.jamcollsurg.2017.07.521. 28739154 10.1016/j.jamcollsurg.2017.07.521 PMC5614873

[119] Maker AV, Katabi N, Qin LX, et al. Cyst fluid interleukin-1beta (IL1beta) levels predict the risk of carcinoma in intraductal papillary mucinous neoplasms of the pancreas. Clin Cancer Res. 2011;17:1502–8. 10.1158/1078-0432.CCR-10-1561. 21266527 10.1158/1078-0432.CCR-10-1561 PMC3065716

[120] Arima K, Okabe H, Hashimoto D, Chikamoto A, Kuroki H, Taki K, et al. The neutrophil-to-lymphocyte ratio predicts malignant potential in intraductal papillary mucinous neoplasms. J Gastrointest Surg. 2015;19(12):2171–7. 10.1007/s11605-015-2973-2. 26443528 10.1007/s11605-015-2973-2

[121] Gemenetzis G, Bagante F, Griffin JF, Rezaee N, Javed AA, Manos LL, et al. Neutrophil-to-lymphocyte ratio is a predictive marker for invasive malignancy in intraductal papillary mucinous neoplasms of the pancreas. Ann Surg. 2017;266(2):339–45. 10.1097/SLA.0000000000001988. 27631774 10.1097/SLA.0000000000001988

[122] McIntyre CA, Pulvirenti A, Lawrence SA, et al. Neutrophil-to-lymphocyte ratio as a predictor of invasive carcinoma in patients with intraductal papillary mucinous neoplasms of the pancreas. Pancreas. 2019;48:832–6. 10.1097/MPA.0000000000001342. 31210665 10.1097/MPA.0000000000001342 PMC7596842

[123] Hata T, Mizuma M, Motoi F, et al. Diagnostic and prognostic impact of neutrophil-to-lymphocyte ratio for intraductal papillary mucinous neoplasms of the pancreas with high-grade dysplasia and associated invasive carcinoma. Pancreas. 2019;48:99–106. 10.1097/MPA.0000000000001202. 30540681 10.1097/MPA.0000000000001202

[124] Zhuge X, Zhou H, Chen L, et al. The association between serum ferritin levels and malignant intraductal papillary mucinous neoplasms. BMC Cancer. 2021;21:1253. 10.1186/s12885-021-08986-z. 34800987 10.1186/s12885-021-08986-z PMC8606075

[125] Serafini S, Friziero A, Sperti C, et al. The ratio of C-reactive protein to albumin is an independent predictor of malignant intraductal papillary mucinous neoplasms of the pancreas. J Clin Med. 2021. 10.3390/jcm10102058. 34064877 10.3390/jcm10102058 PMC8150937

[126] de Visser KE, Joyce JA. The evolving tumor microenvironment: from cancer initiation to metastatic outgrowth. Cancer Cell. 2023;41:374–403. 10.1016/j.ccell.2023.02.016. 36917948 10.1016/j.ccell.2023.02.016

[127] Bullman S. The intratumoral microbiota: from microniches to single cells. Cell. 2023;186:1532–4. 10.1016/j.cell.2023.03.012. 37059062 10.1016/j.cell.2023.03.012

[128] Wei MY, Shi S, Liang C, et al. The microbiota and microbiome in pancreatic cancer: more influential than expected. Mol Cancer. 2019;18:97. 10.1186/s12943-019-1008-0. 31109338 10.1186/s12943-019-1008-0 PMC6526613

[129] Del Castillo E, Meier R, Chung M, Koestler DC, Chen T, Paster BJ, et al. The microbiomes of pancreatic and duodenum tissue overlap and are highly subject specific but differ between pancreatic cancer and noncancer subjects. Cancer Epidemiol Biomarkers Prev. 2019;28(2):370–83. 10.1158/1055-9965.EPI-18-0542. 30373903 10.1158/1055-9965.EPI-18-0542 PMC6363867

[130] Geller LT, Barzily-Rokni M, Danino T, et al. Potential role of intratumor bacteria in mediating tumor resistance to the chemotherapeutic drug gemcitabine. Science. 2017;357:1156–60. 10.1126/science.aah5043. 28912244 10.1126/science.aah5043 PMC5727343

[131] Daniel N, Farinella R, Belluomini F, Fajkic A, Rizzato C, Souček P, et al. The relationship of the microbiome, associated metabolites and the gut barrier with pancreatic cancer. Semin Cancer Biol. 2025;112:43–57. 10.1016/j.semcancer.2025.03.002. 40154652 10.1016/j.semcancer.2025.03.002

[132] Gaiser RA, Halimi A, Alkharaan H, et al. Enrichment of oral microbiota in early cystic precursors to invasive pancreatic cancer. Gut. 2019;68:2186–94. 10.1136/gutjnl-2018-317458. 30872392 10.1136/gutjnl-2018-317458 PMC6872446

[133] Hozaka Y, Oi H, Satake S, et al. Are intratumoral microbiota involved in the progression of intraductal papillary mucinous neoplasms of the pancreas? Surgery. 2023;173:503–10. 10.1016/j.surg.2022.10.003. 36404180 10.1016/j.surg.2022.10.003

[134] Nejman D, Livyatan I, Fuks G, et al. The human tumor microbiome is composed of tumor type-specific intracellular bacteria. Science. 2020;368:973–80. 10.1126/science.aay9189. 32467386 10.1126/science.aay9189 PMC7757858

[135] Baba Y, Tajima K, Yoshimura K. Intestinal and esophageal microbiota in esophageal cancer development and treatment. Gut Microbes. 2025;17:2505118. 10.1080/19490976.2025.2505118. 40376843 10.1080/19490976.2025.2505118 PMC12087659

[136] Zhang T, Li Y, Zhai E, Zhao R, Qian Y, Huang Z, et al. Intratumoral *Fusobacterium nucleatum* recruits tumor-associated neutrophils to promote gastric cancer progression and immune evasion. Cancer Res. 2025;85(10):1819–41. 10.1158/0008-5472.CAN-24-2580. 39992708 10.1158/0008-5472.CAN-24-2580 PMC12079103

[137] Pust MM, Rocha Castellanos DM, Rzasa K, Dame A, Pishchany G, Assawasirisin C, et al. Absence of a pancreatic microbiome in intraductal papillary mucinous neoplasm. Gut. 2024;73(7):1131–41. 10.1136/gutjnl-2023-331012. 38429112 10.1136/gutjnl-2023-331012 PMC11187374

[138] Eckhoff AM, Fletcher AA, Kelly MS, Dohlman AB, McIntyre CA, Shen X, et al. Comprehensive assessment of the intrinsic pancreatic microbiome. Ann Surg. 2024;282(6):1060–9. 10.1097/SLA.0000000000006299. 38623754 10.1097/SLA.0000000000006299 PMC11480254

[139] Fukuda K, Hozaka Y, Oi H, Tomita M, Idichi T, Yamasaki Y, et al. Association between intra-tumoral microbiome and genetic alterations in intraductal papillary mucinous neoplasms of the pancreas. Pancreatology. 2025;25(6):905–11. 10.1016/j.pan.2025.08.001. 40784822 10.1016/j.pan.2025.08.001

[140] Zou L, Mo S, Jia C, Pang J, Chang X, Chen J. The tumoral microbiome of pancreatic intraductal papillary mucinous neoplasm: a single-center retrospective cohort study. J Gastroenterol Hepatol. 2024;39(3):496–505. 10.1111/jgh.16437. 38111357 10.1111/jgh.16437

[141] Sun Z, Huang S, Zhu P, Tzehau L, Zhao H, Lv J, et al. Species-resolved sequencing of low-biomass or degraded microbiomes using 2bRAD-M. Genome Biol. 2022;23(1):36. 10.1186/s13059-021-02576-9. 35078506 10.1186/s13059-021-02576-9 PMC8789378

[142] Pollini T, Adsay V, Capurso G, et al. The tumour immune microenvironment and microbiome of pancreatic intraductal papillary mucinous neoplasms. Lancet Gastroenterol Hepatol. 2022;7:1141–50. 10.1016/S2468-1253(22)00235-7. 36057265 10.1016/S2468-1253(22)00235-7 PMC9844533

[143] Clark CE, Hingorani SR, Mick R, et al. Dynamics of the immune reaction to pancreatic cancer from inception to invasion. Cancer Res. 2007;67:9518–27. 10.1158/0008-5472.CAN-07-0175. 17909062 10.1158/0008-5472.CAN-07-0175

[144] Roth S, Zamzow K, Gaida MM, Heikenwälder M, Tjaden C, Hinz U, et al. Evolution of the immune landscape during progression of pancreatic intraductal papillary mucinous neoplasms to invasive cancer. EBioMedicine. 2020;54:102714. 10.1016/j.ebiom.2020.102714. 32259711 10.1016/j.ebiom.2020.102714 PMC7132171

[145] Hernandez S, Parra ER, Uraoka N, et al. Diminished immune surveillance during histologic progression of intraductal papillary mucinous neoplasms offers a therapeutic opportunity for cancer interception. Clin Cancer Res. 2022;28:1938–47. 10.1158/1078-0432.CCR-21-2585. 35491652 10.1158/1078-0432.CCR-21-2585 PMC9069801

[146] Jamouss KT, Damanakis AI, Cornwell AC, Jongepier M, Trujillo MA, Pflüger MJ, et al. Tumor immune microenvironment alterations associated with progression in human intraductal papillary mucinous neoplasms. J Pathol. 2025;266(1):40–50. 10.1002/path.6402. 40001347 10.1002/path.6402 PMC13014305

[147] Enzler T, Shi J, McGue J, et al. A comparison of spatial and phenotypic immune profiles of pancreatic ductal adenocarcinoma and its precursor lesions. Int J Mol Sci. 2024. 10.3390/ijms25052953. 38474199 10.3390/ijms25052953 PMC10932200

[148] Öhlund D, Handly-Santana A, Biffi G, Elyada E, Almeida AS, Ponz-Sarvise M, et al. Distinct populations of inflammatory fibroblasts and myofibroblasts in pancreatic cancer. J Exp Med. 2017;214(3):579–96. 10.1084/jem.20162024. 28232471 10.1084/jem.20162024 PMC5339682

[149] Chang DZ, Ma Y, Ji B, Wang H, Deng D, Liu Y, et al. Mast cells in tumor microenvironment promotes the in vivo growth of pancreatic ductal adenocarcinoma. Clin Cancer Res. 2011;17(22):7015–23. 10.1158/1078-0432.CCR-11-0607. 21976550 10.1158/1078-0432.CCR-11-0607 PMC4089502

[150] Eckhoff AM, Fletcher AA, Landa K, et al. Multidimensional immunophenotyping of intraductal papillary mucinous neoplasms reveals novel T cell and macrophage signature. Ann Surg Oncol. 2022;29:7781–8. 10.1245/s10434-022-12157-0. 35831529 10.1245/s10434-022-12157-0 PMC9949893

[151] Tanaka M. Intraductal papillary mucinous neoplasm of the pancreas as the main focus for early detection of pancreatic adenocarcinoma. Pancreas. 2018;47:544–50. 10.1097/MPA.0000000000001047. 29702531 10.1097/MPA.0000000000001047

[152] Yamaguchi K, Nakamura K, Yokohata K, et al. Pancreatic cyst as a sentinel of in situ carcinoma of the pancreas. Report of two cases. Int J Pancreatol. 1997;22:227–31. 10.1007/BF02788389. 9444555 10.1007/BF02788389

[153] Tanaka M, Yokohata K, Konomi H, Yamaguchi K, Chijiiwa K, Ohta M. Segmental balloon cytology for preoperative localization of in situ pancreatic cancer. Gastrointest Endosc. 1997;46(5):447–9. 10.1016/s0016-5107(97)70039-7. 9402120 10.1016/s0016-5107(97)70039-7

[154] Yoshioka T, Shigekawa M, Ikezawa K, et al. The relationship between observation interval and prognosis in pancreatic cancer concomitant with intraductal papillary mucinous neoplasia. Pancreatology. 2024;24:73–7. 10.1016/j.pan.2023.11.005. 37996267 10.1016/j.pan.2023.11.005

[155] Matthaei H, Norris AL, Tsiatis AC, Olino K, Hong SM, dal Molin M, et al. Clinicopathological characteristics and molecular analyses of multifocal intraductal papillary mucinous neoplasms of the pancreas. Ann Surg. 2012;255(2):326–33. 10.1097/SLA.0b013e3182378a18. 22167000 10.1097/SLA.0b013e3182378a18 PMC3534752

[156] Pergolini I, Sahora K, Ferrone CR, et al. Long-term risk of pancreatic malignancy in patients with branch duct intraductal papillary mucinous neoplasm in a referral center. Gastroenterology. 2017;153:1284-94.e1. 10.1053/j.gastro.2017.07.019. 28739282 10.1053/j.gastro.2017.07.019

[157] Assawasirisin C, Fagenholz P, Qadan M, Hernandez-Barco Y, Aimprasittichai S, Kambadakone A, et al. Unraveling the long-term natural history of branch duct intraductal papillary mucinous neoplasm: beyond 10 years. Ann Surg. 2025;281(1):154–60. 10.1097/SLA.0000000000006535. 39253809 10.1097/SLA.0000000000006535

[158] Ohtsuka T, Maguchi H, Tokunaga S, et al. Prospective multicenter surveillance study of branch-duct intraductal papillary mucinous neoplasm of the pancreas; risk of dual carcinogenesis. Pancreatology. 2024;24:1141–51. 10.1016/j.pan.2024.08.013. 39191596 10.1016/j.pan.2024.08.013

[159] Oyama H, Tada M, Takagi K, et al. Long-term risk of malignancy in branch-duct intraductal papillary mucinous neoplasms. Gastroenterology. 2020;158:226-37.e5. 10.1053/j.gastro.2019.08.032. 31473224 10.1053/j.gastro.2019.08.032

[160] Ideno N, Ohtsuka T, Matsunaga T, et al. Clinical significance of GNAS mutation in intraductal papillary mucinous neoplasm of the pancreas with concomitant pancreatic ductal adenocarcinoma. Pancreas. 2015;44:311–20. 10.1097/MPA.0000000000000258. 25479586 10.1097/MPA.0000000000000258

[161] Oi H, Hozaka Y, Akahane T, et al. Genetic assessment of intraductal papillary mucinous neoplasm for predicting concomitant pancreatic ductal adenocarcinoma. Pancreas. 2024;53:e790–5. 10.1097/MPA.0000000000002373. 38743932 10.1097/MPA.0000000000002373

[162] Oyama H, Hamada T, Nevo D, et al. Relationship of intrapancreatic fat deposition with pancreatic cancer differs according to carcinoma types. Gastroenterology. 2025;169:718-21.e5. 10.1053/j.gastro.2025.04.032. 40446943 10.1053/j.gastro.2025.04.032

[163] Truong E, Pandol S, Jeon C. Uniting epidemiology and experimental models: pancreatic steatosis and pancreatic cancer. EBioMedicine. 2022;79:103996. 10.1016/j.ebiom.2022.103996. 35405390 10.1016/j.ebiom.2022.103996 PMC9010750

[164] Takahashi K, Takeda Y, Ono Y, et al. Current status of molecular diagnostic approaches using liquid biopsy. J Gastroenterol. 2023;58:834–47. 10.1007/s00535-023-02024-4. 37470859 10.1007/s00535-023-02024-4 PMC10423147

[165] Yachida S, Yoshinaga S, Shiba S, et al. KRAS mutations in duodenal lavage fluid after secretin stimulation for detection of pancreatic cancer. Ann Surg. 2025. 10.1097/sla.0000000000006645. 39902566 10.1097/SLA.0000000000006645 PMC13557476

